# M. Virey’s Refutation of Liebig’s Theory of the Uses of the Respiration and of Food

**Published:** 1842-11-12

**Authors:** J. J. Virey


*Refutation of Liebig's theory of the uses of the Respiration and of Food.* By M. J. J. Virey.—It may be recollected, that Liebig holds that the chief use of the food is to supply carbon and hydrogen, which, uniting with the oxygen absorbed from the air, give rise to the generation of animal heat. He consequently holds that there is a certain fixed relation between the amount of food consumed, and the quantity of carbon and hydrogen thrown off at the lungs. M. Virey opposes this theory, as contrary to common observation, as, even though it be allowed to be applicable to mammalia, birds, and reptiles, it is by no means to those animals which respire by means of branchiæ. Thus all animals with the branchiæ consume but little oxygen, comparatively speaking, and yet many of them devour very great quantities of food. Even the largest and most voracious of the reptiles, as the alligators, crocodiles, &c., which devour enormous quantities of food, under a burning climate too, respire feebly with their vesicular lungs, and consume but little oxygen.

Fishes, whose blood is but imperfectly oxygenated by the branchial apparatus, are perhaps among the most voracious of animals, and yet, according to Liebig's theory, they ought to eat little, because they consume little oxygen.

The same holds true of the Mollusca. The cuttle-fish, ***buccinum, strombus, murex***, &c. grow to a large size; but their respiration is very imperfect, and yet they are great flesh-eaters. The Crustacea, again, as the crabs, lobsters, &c. grow rapidly, because they are great eaters; but their branchial apparatus is not fitted to consume much oxygen.

In all these animals assimilation takes place very rapidly, notwithstanding their feeble respiratory powers; and they are, besides, by no means deficient in activity or muscular powers, though, their flesh be but feebly azotized or animalized, and their blood is always cold.

If it be one of the characters of vitality, that the more perfect this principle is the greater is the number of germs, or eggs, or fœtuses produced, then, quite contrary to Leibig's theory, the number of germs produced is in the inverse ratio of the perfection of the respiratory functions. Fishes and mollusca deposit their spawn or eggs by millions; but the mammalia, and even the birds, whose respiratory functions are the most perfect, are in this respect infinitely behind these. On the other hand, it is seen that the number of germs or eggs is rather proportioned to the nutrition received; for the amount of food taken is not proportioned to the respiration in the animal kingdom.

M. Virey, therefore, concludes, that the vital force or central nervous energy has more to do with the production of animal heat than the consumption of carbon at the lungs, and this for three special reasons; 1st, Because a fecundated egg resists a freezing temperature longer than one which has not been fecundated. 2d, That a hybernating insect, reptile, or animal, or even trees during winter, by the sole influence of a vital power, resist a freezing temperature, whereas the same animals, if dead, would be instantly frozen. 3d, That many mammalia and birds keep themselves warm even in the most rigorous winters under the Pole, not in consequence of a greater amount of oxygen consumed, nor by a greater amount of muscular activity, but in consequence of a more abundant highly azotized or animalized nourishment.—*Ibid, from Journal de Pharmacie*, May 1842.

---

*Academy of Medicine, Paris, August* 30.

*Vaccination.*—M. Bousquet read a report on a memoir of M. Milon, entitled "Remarks on Vaccination." The author had occasion to observe an epidemic of small-pox and test the protective power of vaccination. The disease was invariably arrested by vaccination, but a few vaccinated persons were attacked; in all these cases the individuals had been vaccinated a long time anteriorly to the attack of small-pox, which, however, always showed itself under the form of varioloid.

It has been frequently said that the protective power of the vaccine matter is only temporary; if by this is meant that varioloid may occur at some period after vaccination, the assertion is true; but not so, if it mean that true small-pox occurs after vaccination. M. Milon does not believe that the efficacy of the vaccine matter has undergone any change from the lapse of time. "I was of the same opinion (says the reporter) up to 1836, but having then witnessed the action of new vaccine matter, I was compelled to acknowledge that the old had lost a portion of its efficacy."

*Microscopic Characters of the Sputa in Consumptive Patients.*—M. Sandras read a memoir on this subject. The sputa were taken from the patients incontestably laboring under phthisis, and examined with a microscope magnifying 300 diameters. The result of the author's investigations is, that the matter expectorated by consumptive patients is of a specific kind, and that the microscope is a most valuable adjunct to our means of diagnosis. The sputa of phthisical patients contains numerous round isolated globules of a grey-white colour, similar in form and size to those of pus, but differing from the latter in this, that whereas pus globules are perfectly free, the former are enclosed in a flocculent envelope, which cannot be washed off from them. To see the globules well, we should avoid collecting too many of them under the field of the microscope. Another character of these globules is their complete opacity in the centre, and their gradual transparency towards the circumference. These facts were observed in forty-nine cases of pulmonary consumption.

The tubercular sputa, however, do not exist in all consumptive patients, and even in the same subject the expectorated matter presents, as we can readily understand, a variety of appearances. A remarkable fact is, that the globules now alluded to are not found in the pus taken from scrofulous glands or tubercular ulcers of the intestinal canal. The author has examined, for the purpose of comparison, the expectoration in simple catarrh. Eighteen patients were examined, but the sputa never contained any globules; they contained corpuscles differing from tubercular globules; first, in being agglomerated and not distinct; second, in being of different sizes; third, in the uncertain manner of their appearance under the field of the microscope; and fourth, in being striated on the surface.

In spite of these differential characters, some cases presented themselves in which the author hesitated; they were chiefly cases where the physical signs and the progress of the disease seemed to contradict the signs furnished by the microscope; but in these doubtful cases it generally happened that the result of the autopsy showed the instrument to have been right. These newly indicated characters may assist and complete our other means of diagnosis. The author does not, however, consider the expectorated matter as being tubercular; on the contrary, he thinks that the pus is secreted by the tissues which surround the tubercles; but he concludes that it acquires from the tubercular matter the peculiar characters which he has indicated.—*Prov. Med. Journ.* Sept. 17, 1842.

---

*Academy of Sciences, Paris, September* 5, 1842.

*New cause of Emphysema.*—M. Louget read a memoir on the nature of the intrinsical motion of the lungs and a new cause of pulmonary emphysema. The following are the conclusions at which the author arrives:—

1. When galvanism is applied to the branches sent from the vagus to the first divisions of the bronchia, it excites evident contractions in these tubes; the animals experimented on should be of lofty stature.

2. Division of the pneumogastric nerves may be followed by emphysema.

3. This seems to contradict the idea that the capillary vesicles of the lung are formed of elastic fibrous tissue.

4. Their parietes are endowed with active contractility, under the influence of the vagus.

5. This contractility ceasing on division of the vagus, it becomes impossible for the air to be renewed in the ultimate branches of the air-tubes, although their elasticity remains intact.

6. Circulation is impeded, or even suspended along the walls of these tubes, which are forcibly distended by vitiated air, containing a large proportion of carbonic acid gas.

7. Emphysema, thus complicated with pulmonary congestion, constitutes a cause of asphyxia.—*Ibid.*

